# Durability and Mechanical Properties of Concrete Reinforced with Basalt Fiber-Reinforced Polymer (BFRP) Bars: Towards Sustainable Infrastructure

**DOI:** 10.3390/polym13091402

**Published:** 2021-04-26

**Authors:** Osama Ahmed Mohamed, Waddah Al Hawat, Mohammad Keshawarz

**Affiliations:** 1 Departent of Civil Engineering, College of Engineering, Abu Dhabi University, Abu Dhabi PO Box 59911, United Arab Emirates; waddah.alhawat@adu.ac.ae; 2 Department of Civil & Biomedical Engineering, College of Engineering, Technology, and Architecture, University of Hartford, West Hartford, CT 06117, USA; keshawarz@hartford.edu

**Keywords:** basalt fiber-reinforced polymers, concrete, reinforcing bars, sustainable construction, durability, bond to concrete

## Abstract

Reducing the fingerprint of infrastructure has become and is likely to continue to be at the forefront of stakeholders’ interests, including engineers and researchers. It necessary that future buildings produce minimal environmental impact during construction and remain durable for as long as practicably possible. The use of basalt fiber-reinforced polymer (BFRP) bars as a replacement for carbon steel is reviewed in this article by examining the literature from the past two decades with an emphasis on flexural strength, serviceability, and durability. The provisions of selected design and construction guides for flexural members are presented, compared, and discussed. The bond of BFRP bars to the surrounding concrete was reportedly superior to carbon steel when BFRP was helically wrapped and sand coated. Experimental studies confirmed that a bond coefficient *k_b_* = 0.8, which is superior to carbon steel, may be assumed for sand-coated BFRP ribbed bars that are helically wrapped, as opposed to the conservative value of 1.4 suggested by ACI440.1R-15. Code-based models overestimate the cracking load for BFRP-reinforced beams, but they underestimate the ultimate load. Exposure to an alkaline environment at temperatures as high as 60 °C caused a limited reduction in bond strength of BFRP. The durability of BFRP bars is influenced by the type of resin and sizing used to produce the bars.

## 1. Introduction

Sustainability and durability of building structures are amongst the leading design criteria for new infrastructure. The contribution of the production of cement to the emission of CO_2_ and environmental pollution prompted the pursuit of alternative cementitious materials, including, but not limited to, fly ash and slag. Partial replacement of cement with fly ash, slag, and/or silica fume has become a common practice and the mechanical properties of such concrete types have been studied extensively. On the other hand, exclusive use of materials such as fly ash and slag as sole cementitious materials activated using carefully selected alkalis has attracted the attention of researchers. Similarly, traditional reinforcing steel bars, despite their favorable mechanical properties, are associated with significant emission of CO_2_ during the manufacturing process, not to mention corrosion that may lead to a loss of cross-section. In the past two decades, interest has been renewed in reinforcing bars made of fiber-reinforced polymers (FRPs). The unidirectional fibers that constitute typically more than 75% by volume are made of glass, carbon, aramid, and, more recently, basalt. Basalt FRP (BFRP) reinforcing steel bars offer numerous favorable properties over traditional reinforcing steel bars, including, but not limited to, high tensile strength, corrosion resistance, and nonmagnetic nature. Studies by [1] concluded that BFRP-reinforced beams have similar global warming potential (GWP) compared to cast-in-place concrete reinforced with 100% recycled carbon steel and, as a result, BFRP-reinforced beams have a limited environmental advantage compared to steel-reinforced concrete beams. On the other hand, concrete beams reinforced with BFRP prestressing bars have much lower GWP compared to beams prestressed with steel bars.

In aggressive environments, traditional reinforcing steel bars are susceptible to corrosion that could influence the structure′s life span. Marine environments and parts of the world where deicing salts must come in contact with concrete are examples of situations where traditional reinforcing steel bars may be subjected to corrosion. It is often less expensive and environmentally friendly to use sea sand for concrete structures that will be in contact with seawater, in which case the noncorroding FRP reinforcing bars would be a durable alternative to traditional steel. The magnetic properties of FRP bars make them suitable for consideration in structural elements surrounding magnetic resonance imaging (MRI) units and any other equipment sensitive to magnetic fields. Sea sand typically contains chloride irons that may cause corrosion of reinforcing steel bars. Design guides for concrete reinforced with FRP bars, such as ACI440.1R-15 [2], were developed and are continuously updated. Most design guides explicitly refer to FRP bars made of glass (GFRP), carbon (CFRP), or aramid (AFRP) fibers, which were studied extensively. However, ACI440.1R makes no explicit reference to concrete-reinforcing FRP bars made of basalt fibers. BFRP bars offer many favorable properties such as high temperature resistance and favorable behavior in an acidic environment, in addition to ease of manufacturing. BFRP reinforcing bars typically fall between CFRP and GFRP bars in terms of strength and stiffness. Studies have shown that GFRP reinforcing bars can be used effectively as corrosion-resistant reinforcement for hollow concrete columns (HCCs) that have many applications, such as bridge piers [3]. Glass fibers were also used successfully in nonbuilding structures such as composite sleepers for railway tracks [4]. GFRP reinforcing bars are susceptible to simulated alkaline environments, resulting in degrading of the fiber–matrix interface [5]. The purpose of this article is to provide a critical review of the literature on the mechanical properties and durability of concrete reinforced with FRP bars made with the promising basalt fibers. This article presents, to practicing engineers, the current state of knowledge on the properties BFRP-reinforced flexural members in support of decision making related to selecting materials for engineering projects. In addition, the article explores the merits and demerits of BFRP in comparison to some of the existing alternatives (e.g., GFRP and CFRP) in terms of strength and durability. Research needed to fill gaps in the knowledge in terms of the durability and strength of BFRP reinforcing bars is identified in this article as well.

## 2. Composition and Properties of Basalt Fiber-Reinforced Polymers (BFRPs)

A basalt FRP bar is a composite material consisting of rigid polymer resin bounding unidirectional basalt fibers. Basalt fibers are produced by melting queried and crushed natural volcanic basalt rocks at a temperature of nearly 1400 °C [6]. The molten rock is extruded through small nozzles to produce continuous filaments of basalt fibers ranging in diameter from 13 to 20 µm. A critical process in the manufacturing of fibers, in general, is known as fiber sizing. Sizing involves the application of a thin layer of mainly organic material known as the size to the surface of the fiber. Most importantly, the short-term and long-term performance of FRP bars is critically influenced by the optimization of the fiber sizing as well as the fiber–matrix interface [7]. The fiber sizing film consists of a film former and a coupling agent. The film former protects, lubricates, and holds the fibers together while ensuring their separation when the fibers come in contact with the resin. The coupling agent, typically an alkoxysilane compound, serves to bond the fibers to the matrix resin [8]. However, the composition and process of applying the fiber size layer vary significantly amongst manufacturers, resulting in variations in properties of FRP bars made of the same type of fiber and sometimes the same resin type.

The resulting composite material, consisting of polymeric resin and fibers, offers numerous favorable properties, including, but not limited to, high tensile strength, with applications in building new structures, such as FRP reinforcing bars, or retrofitting/strengthening deficient existing structures using FRP sheets and/or strips [9].

BFRP bars are commonly manufactured through the pultrusion process, which involves pulling the continuous fibers through a die that is circular in cross-section and contains resin. The FRP bars are formed once the resin cures (thermosets) in the die. The amount of basalt fiber in BFRP bars is not standardized, but the fiber content most frequently reported in the literature falls in the range 75% to 90% [10,11]. Automated wet-layup is another method to manufacture BFRP bars that reportedly offers the same degree of variation in mechanical properties as the pultrusion process [12]. As the resin has much lower strength compared to the fibers, the tensile strength and stiffness of BFRP bars varies depending on the overall volume of fibers to volume of FRP. Vinyl ester and isophthalic polyester are common types of resin matrix used to manufacture BFRP.

FRP bars are more sensitive to fire than steel bars. However, because the FRP bars are embedded in concrete, they do not contribute to fire severity nor toxicity. Nonetheless, FRP-reinforced concrete elements have lower resistance to fire compared to steel-reinforced concrete elements [13]. More importantly, at temperatures close to the glass transition temperature of the polymer, T_g_, mechanical properties of the polymer deteriorate, and its ability to transfer stresses between the fiber and the surrounding concrete decreases [14]. The structural implication is the degrading of the bond strength between FRP bars and concrete. Glass transition temperatures for most resins used to manufacture FRP reinforced bars range from 93 °C to 120 °C.

**Figure 1:**
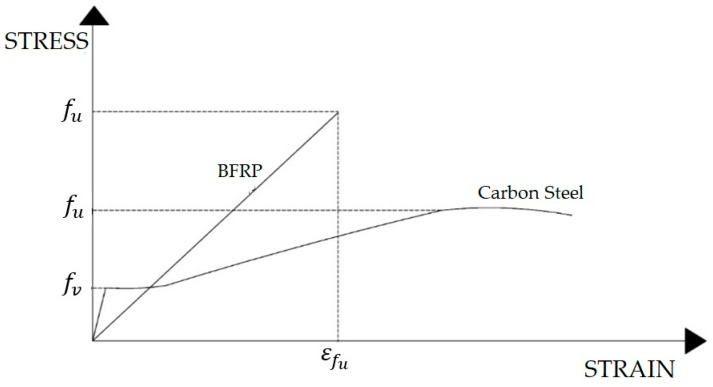
Typical stress–strain relationship of carbon steel and basalt fiber-reinforced polymer (BFRP) bars.

BFRP bars may be 2.3 times stronger or more , in terms of ultimate strength (f_u_), than traditional steel reinforcing. However, the modulus of elasticity of traditional steel may be 3.5 times or greater than BFRP. BFRP elastic moduli varying from 44.5 to 71 MPa were reported in the literature [11,15,16], depending on resin type, manufacturer, and sometimes bar diameter. Unlike traditional carbon steel, FRP bars do not exhibit yielding, as shown in Figure 1. Tensile strength reported in the literature varied from 1100 to 1565 [11,15,16]. These wide ranges of values for the tensile strength and modulus of elasticity were reported for BFRP produced by different manufacturers, which not only reflect variation in the properties of resin but also manufacturing. Nonetheless, variability in moduli and strength were reported in BFRP bars produced by the same manufacturer, although with less dispersion. In comparison, due the homogeneity of steel, the modulus of elasticity of and tensile strength can largely be assumed to be constant for all practical purposes.

**Figure 2:**
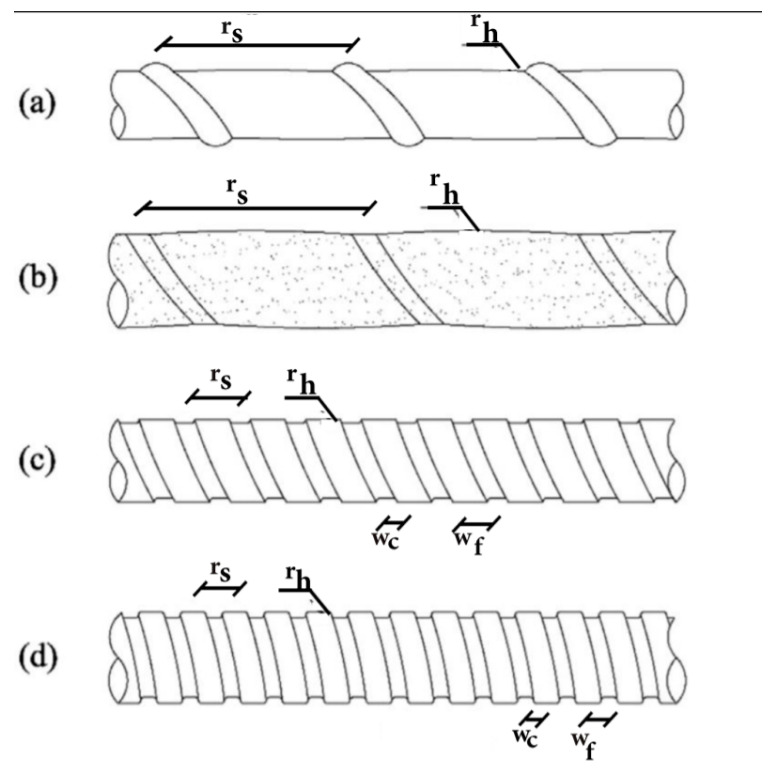
Fiber-reinforced polymers (FRP) bar surface configurations: (**a**) Ribbed and helically wrapped, (**b**) ribbed, helically wrapped, and sand coated, (**c**) indented, (**d**) ribbed.

It is to be expected that the external surface configuration of BFRP bars affects the effectiveness of bonds to the surrounding concrete. The external surface may be helically wrapped with fibers, as shown in Figure 2, with or without additional sand coating. BFRP bars may also have deformations (ribs or indents) without helical fiber wrapping, with or without sand coating. The most common are ribbed BFRP bars with helical fiber wrapping and sand coating. Kevlar fibers (0.4 mm in diameter) are often used for helical wrapping [17]. Figure 2 shows schematics of various FRP bar configurations. Ribbed and helically wrapped BFRP bars that are sand coated provide the highest bond strength, as will be discussed later in this article. Nonetheless, the method of sand coating appears to also affect the bond strength although no standardized method of sand coating is available. Sand coating of FRP reinforcing bars was used before the advent of BFRP bars to enhance bond strength and was proven to enhance bond strength [18]. It is typical to apply the helically wound fibers and sand coating after the pultrusion process, but before the thermosetting of the polymeric resin [19].

As a result of a lack of standardization, the increase in BFRP bar area due to sand coating is inconsistent and may vary with bar diameter, even when produced by the same manufacturer [20]. Similarly, tensile strength and modulus of elasticity also varied from one bar size to another in BFRP obtained from the same source. For wrapped BFRP bars without sand coating, variations in bond strength were also reported based on rib size and rib spacing [21], but further studies are needed to quantify the observation. BFRP bars with woven surfaces (no ribs) are manufacturing for use as prestressing bars, with lower bond strength compared to ribbed bars [22]. 

## 3. Bond of BFRP Bars to Concrete

The ability of concrete flexural members to sustain applied loads is highly dependent on the bond characteristics between basalt fiber-reinforced polymer (BFRP) and the surrounding concrete. Specifications for BFRP bars are not explicitly mentioned in ACI440.1R-15 [2]. However, CSA [23] specifies a minimum bond strength of 8 MPa for BFRP bars. Much higher bond strength was reported in the literature for most BFRP bar surface configurations/preparations.

Henin et al. [24] noted that fatigue loads decrease bond stiffness and increase slip for different bar–rib configurations, compared to static loads. However, the phenomenon depends on the fatigue stress level. The higher the fatigue stress level, the higher the bond stiffness. The investigators noted a slight improvement in bond strength under fatigue load compared to a static load. On the other hand, fatigue load aggravates the bond interface damage. Bond characteristics reported in the literature include studies on different FRP bar configurations such as ribbed/indented, ribbed/indented/helically wrapped without sand coating, and ribbed/indented/helically wrapped but without sand coating. The optimum bond strength was obtained when BFRP bars were ribbed and sand coated along the bar length [22]. The effect of combined helical wrapping and sand coating reportedly produces the best bond characteristics, except for the study by Solym and Balazs [22], which reported sand coating of ribbed bars to produce better bond characteristics than combining sand coating and helical wrapping of bars.

Several guides, standards, and codes developed test procedures to determine bond strength that was used to develop bond–slip relationships [23,25,26]. The bar pull-out test is the most common amongst all standards and guides. 

The bond strength, *τ* (MPa, psi), is related to the tension force, *F* (N, Ibf), through Equation (1).

(1) $$\tau =\frac{F}{C_{b}\;l}$$

where: *τ* = average bond stress, MPa (psi); *F* = tensile force, N (lbf);

*C_b_* = effective circumference of FRP bar, calculated as 3.1416 *d_b_* where *d_b_* is the effective bar diameter of the bar, calculated according to Test Method D7250/D7250M, mm (in);
*l* = bonded length, mm (in).

Typically, the failure load is influenced by the bond length, *l*, which is defined as the length in contact with concrete.

### 3.1. Effect of Rib Spacing and Rib Depth of BFRP Reinforcing Bars on Bond Strength

**Figure 3:**
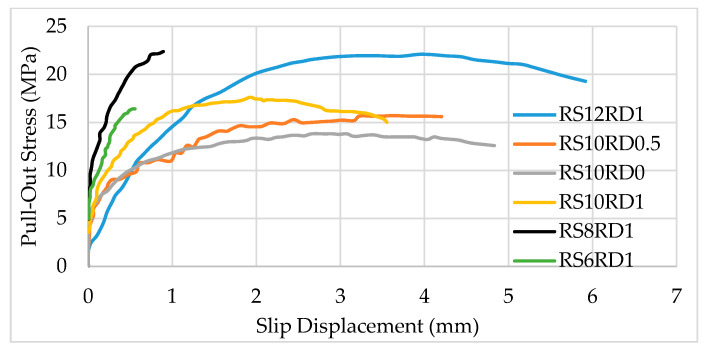
Pull-out test result showing stress versus slip for 10 mm diameter BFRP bars with bond length *l* = 5D with various rib spacings and rib depths. RS12RD1 = Rib spacing of 12 mm and rib depth of 1 mm. Reprinted with permission from ref. [21]. Copyright 2021 ASTM International.

Studies by [21] concluded that rib spacing and rib depth of BFRP bars affect the bond strength. Figure 3 shows a bond–slip relationship for 10 mm BFRP bars with rib spacing of 6, 8, 10, and 12, and rib depth of 0 mm, 0.5 mm, or 1 mm (RS8RD1 means rib spacing of 8 mm and rib depth of 1 mm). Generally speaking, for bars with the same rib spacing (e.g., RS10), the larger the rib depth, the higher the bond strength (RS10RD1 exhibited higher strength compared to RS10RD0.5 and RS10RD0). There are three mechanisms for the bond between BFRP bars and concrete, namely, chemical adhesion, mechanical interlocking, and friction. Friction bonding is particularly significant when BFRP bars are coated with sand [11]. For loads up to 20% of the maximum load, the bond is mostly due to chemical adhesion, and there is very little movement at the load end and no displacement at the free end regardless of the rib spacing or rib depth [21]. Movement at the free end begins at roughly 40% of the maximum load and the strength–slip relationship becomes nonlinear. When the maximum load is reached, shear fracture occurs at the interface between the BFRP and surrounding concrete.

**Figure 4:**
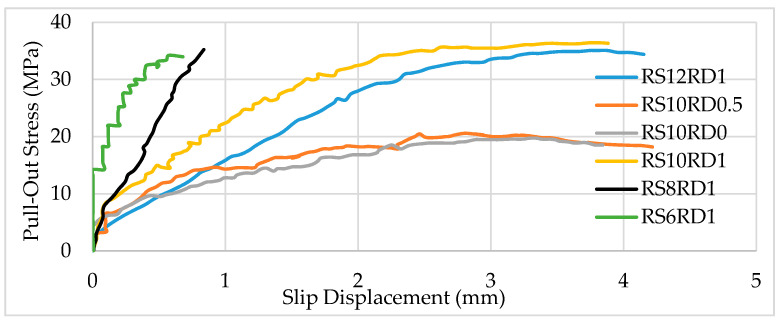
Pull-out stress showing stress versus slip for 10 mm diameter BFRP bars, with *l* = 10D and various rib spacings (RS) and rib depths (RD).

The maximum bond strength increases with an increase in bond length for bars of the same material, diameter, rib spacing, and rib depth. Figure 4 shows the stress–slip relationship for *l* = 10D. Comparing Figure 4, it can be seen, for example, that bond strength for RS12RD1 was 21 MPa with *l* = 5D, which is less than the strength of the same bar (33 MPa) when *l* = 10D.

### 3.2. Bond Strength–Slip Relationship

**Figure 5:**
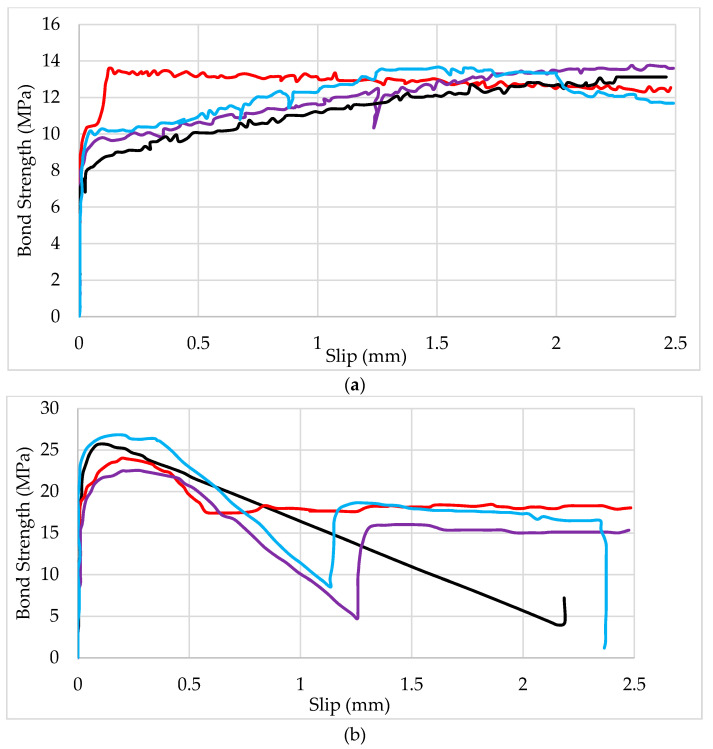
Bond strength–slip relationship for 10-mm diameter wrapped samples (each color is different sample) BFRP bars with (**a**) primary sand coating, (**b**) secondary sand coating. Reprinted with permission from ref. [27]. Copyright 2021 Elsevier Science & Technology Journals.

The relationship between bond strength and slippage between BFRP and the surrounding concrete is essential to the design of flexural members. As indicated earlier, the effectiveness of the bond strength depends on several factors, including, but not limited to, surface configuration/preparation of the BFRP bars. Several studies have shown that ribbed/indented, helically wrapped, and sand-coated BFRP provides the highest bond strength compared to BFRP without wrapping or without sand coating [27]. One study, however, reported better bond strength for sand-coated ribbed FRP bars without helical wrapping compared to sand-coated bars with helical wrapping [22]. It is worth noting that the latter study showed a greater variety of results from one sample to another and samples were provided by different manufacturers. The first phase of the bond strength–slip relation is linear, corresponding to a small slippage, which is attributed to chemical adhesion. For wrapped BFRP bars with primary sand coating, the linear phase is followed by a nonlinear phase up to the peak bond strength. No significant increase in bond strength occurs after the peak until complete slippage failure occurs (Figure 5a). When wrapped BFRP bars are treated with a secondary sand coating, the peak bond strength is much larger than the bars with primary sand coating, as shown in Figure 5b. The peak strength is followed by a significant drop in bond strength due to partial failure of the secondary sand coating layer. The primary sand coating layer causes an increase in bond strength again (third phase), as shown in Figure 5b, up to a peak bond strength which then remains essentially constant until complete bond failure occurs [27].

When wrapped BFRP reinforcing bars are not coated with sand, the bond strength is affected by rib depth (half of the difference between the outer diameter including the ribs and inner diameter excluding the rib), rib spacing, and rib configuration (spacing and depth/height affect the mechanical interlock phase of the total bond strength). Therefore, large rib spacing (or lack thereof) leads to a much weaker bond strength of BFRP bars compared to unribbed bars, regardless of sand coating [21]. The sand coating enhances bond strength through a contribution to both the friction component and enhancing mechanical interlocking.

### 3.3. Bond Coefficient and Flexural Crack Control of BFRP-Reinforced Concrete Flexural Members

Similar to traditional carbon steel, the characteristics of the bond between FRP reinforcing bars and the surrounding concrete affects crack width under service load. The current philosophy for controlling service load crack width, *w*, in ACI318 was also adopted in ACI440.1R [2], which relies on controlling the spacing between the reinforcing bars. Equation (2) expresses the maximum spacing between tension reinforcing FRP bars. Definitions of all parameters for the equations appearing in this article are listed in Abbreviations.

(2) $$S_{max}=1.15\;\frac{E_{f}w}{f_{fs}k_{b}}-2.5C_{c}\;\leq 0.92\;\frac{E_{f}w}{f_{fs}k_{b}}$$

where:

*E_f_* = design or guaranteed modulus of elasticity of FRP defined as mean modulus of sample of test specimen (*E_f_* = *E_average_*), MPa (psi);
*f_fs_* = stress level induced in FRP at service load, MPa (psi);
*C_c_* = clear cover, mm (in);
*k_b_* = bond-dependent coefficient.

The experimental bond coefficient is *k_b_* = 1.0 when FRP bars have the same bond characteristics as carbon steel, while a bond coefficient less than 1.0 indicates a superior bond between FRP bars and concrete in comparison to steel. On the other hand, a bond coefficient greater than 1.0 indicates that the FRP bond performance is inferior to traditional steel. Studies on various fiber manufacturers, cross-sections, and resin types indicate that the bond coefficient could vary from 0.6 to 1.72 [2]. ACI440.1R recommends a value of *k_b_* = 1.4 to be used in the absence of test data. The variation in the type of fiber (carbon, aramid, glass, and basalt) contributes to the reported wide range of *k_b_* values.

The recommended bond coefficient for sand-coated, fiber-wrapped BFRP bars in CSA [23] is *k_b_* = 0.8, indicating the superior performance of BFRP bars compared to steel.

Calculation of the bond coefficient is commonly obtained from the crack width model in CSA and is represented by Equation (3).

(3) $$w=2\frac{F_{f}}{E_{f}}\;\beta k_{b}\;\sqrt{{\left(\frac{s}{2}\right)}^{2}+d_{c}{}^{2}}$$

where:

w = maximum on the tension side;
*E_f_* = modulus of elasticity of FRP;
*F_f_* = flexural stress in FRP bars;
*β* = ratio of the distance from the neutral axis to extreme tension fiber to distance from neutral axis to center of tensile reinforcement;
*d_c_* = thickness of concrete cover measured from extreme tension fiber to center of tension bars
*s* = spacing of longitudinal bars.

Four-point loading tests on concrete beams reinforced with deformed, sand-coated, and wrapped BFRP bars indicate superior bond performance, with *k_b_* = 0.76 [11]. This study was conducted on 2.7 m clear span simply supported beams prepared using 42.5 MPa concrete compressive strength, and reinforced with 10, 12, and 16 mm diameter BFRP bars.

Experimental studies by [27] deduced a bond coefficient *k_b_* = 0.77 for double sand coating of wrapped BFRP bars and *k_b_* = 0.92 for single sand coating. Clearly the bond coefficient of double sand coating is close to the recommended CSA [23] value but far less than 1.4 recommended by [2]. The calculation of the bond coefficient in this study was based on the assumed crack width of 0.7 mm, which is the upper limit of the crack width indicated for aesthetic purposes in [2]. 

The bond coefficient for any type of bundled BFRP bars is higher than individual bars. The reduced bond effectiveness in bundled BFRP bars, as indicated by the higher *k_b_* value, is due to the reduced area in contact with concrete. This is consistent with the requirement for longer development length when traditional carbon reinforcing steel is bundled. *k_b_* = 1.25 was recommended for wrapped BFRP bars that are sand coated [27].

The bond coefficient is often calculated based on controlled crack width, as indicated earlier. It may also be determined based on estimated service load which is sometimes taken as the load causing a stress level of 0.25$f_{fu}$ ($f_{fu}$ = ultimate tensile strength of BFRP) or 0.3$M_{n}$ ($M_{n}=$ nominal flexural capacity). Studies indicate that *k_b_* calculated based on service load is lower than the values calculated based on controlled crack width [27].

### 3.4. Effect of Strain Rate on Bond Strength

Structures may be subjected to dynamic loads, such as those caused by earthquakes or blasts, which are often simulated experimentally by applying strains at a faster rate. Studies on 10 mm diameter BFRP bars showed that bond strength increases while slip decreases with increasing strain rate. The strain rate included in the study ranged from 3.68 × 10^−4^ s^−1^ (simulating static loading) to 3.68 × 10^−1^ s^−1^ [28]. However, studies by [29] show that the bond strength of BFRP bars in concrete made with sea sand decreases with an increase in loading rate. The study covered test machine strain rates ranging from 6.4 × 10^−5^ s^−1^ (simulating static loading) to 51.3 s^−1^ (simulating impact). Sea sand is often used to diversify the use of natural sand resources, and it is often the least expensive alternative structure in contact with seawater.

### 3.5. Effect of Temperature on Bond Strength

A legitimate concern on the use of FRP reinforcing bars is their performance under elevated temperatures. While basalt fibers are naturally fire resistant, the resin that binds the fibers together cannot withstand elevated temperatures. In general, the bond strength between FRP bars and concrete decreases with an increase in temperature. However, BFRP bars maintained better bond strength compared to GFRP bars at elevated temperatures ranging from 70 °C to 220 °C [30]. The loss of bond strength at 220 °C of BFRP bars was 7.11% compared to the bond strength of the bars at room temperature. However, at 270 °C, BFRP bars lost nearly 32% of their bond strength, but at 350 °C, the loss in bond strength was significantly higher [30].

## 4. Flexural Response and Contribution to Compression Forces

Longitudinal GFRP and CFRP reinforcing bars used in columns contributed as little as 5% to 12% of the ultimate axial compression capacity, therefore, ACI440.1R recommends neglecting all contributions to compression in columns as well as beams [2]. Studies have shown that equivalent columns reinforced with either GFRP or BFRP have largely the same capacity and respond similarly [31], which is not surprising as the response is likely dominated by the resin and resin–fiber interface. Some studies reported the BFRP contribution to be as high as 24% of the axial ultimate compression capacity when 7% BFRP bars are used as primary longitudinal reinforcement when steel ties are used [31]. The contribution of BFRP reinforcement to the ultimate compression capacity is influenced by the amount of reinforcement, the shape of the column cross-section, concrete strength, and type of ties (material, strength, and spacing). Despite concerns over the effect of creep on strength of FRP bars in general, the contribution of BFRP bars to compression capacity deserves further investigations and need not be completely discounted.

In the sequent subsections of this article, the discussion is limited to the response of flexural members reinforced with BFRP bars placed on the tension side as is typically the case in beams. The discussion in this article generally applies to the flexural response of two-way floor slabs reinforced with FRP bars. However, some experimental studies and reviews also showed FRP bars with a promising ability to resist two-way shear (punching shear) in concrete flat slab/plate, although further research is needed to understand failure modes and quantify limitations [32].

### 4.1. Ultimate Load and Cracking Pattern

**Figure 6:**
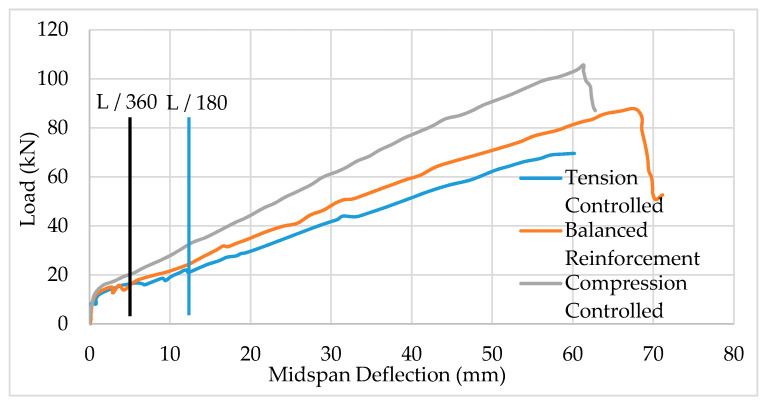
Load–deflection relationship for beams reinforced with BFRP bars and BFRP stirrups Reprinted with permission from ref. [16]. Copyright 2021 American Society of Civil Engineers.

Flexural members reinforced with BFRP bars exhibit many similarities to the response of beams reinforced with carbon steel. As indicated earlier in this article, the modulus of elasticity of BFRP bars (44 to 72 MPa) is much lower than the typical modulus of carbon steel (200 MPa), which influences flexural response. Load–deflection tests of BFRP-reinforced beams showed that stiffness remains essentially the same until cracking begins, regardless of reinforcement ratio, and whether or not the beams have shear reinforcement [16]. As shown in Figure 6, the load–deflection relationship of BFRP-reinforced beams remains largely linear until cracking, whether the beams are under-reinforced, balanced, or over-reinforced. After cracking, the stiffness decreases as expected, but the load–deflection relationship remains linear until failure, which may occur due to stirrup rupture when BFRP stirrups are used, as shown in Figure 6 [16]. Over-reinforced BFRP beams are stiffer than tension-controlled and balanced BFRP concrete beams. Therefore, they experience slightly less deflection under the same service load, compared to balanced and under-reinforced beams. For example, at a deflection limit of L/180, it is clear that the compression-controlled beam (over-reinforced) carries more load compared to the balanced and tension-controlled beams.

For beams loaded to failure in flexure, the crack width and distribution were enhanced by increasing the reinforcement ratio. This enhanced response was attributed to increased stiffness with increased BFRP reinforcement. Crack width, however, is not a durability concern in BFRP-reinforced beams as the bars do not corrode, unlike steel-reinforced beams. Crack width is only an aesthetic concern rather than a safety problem. Therefore, for aesthetic considerations, a larger crack width of 0.5 mm is permitted for exterior exposure while 0.7 mm is permitted for interior exposure [23].

Experimental studies by [15] show that the ultimate moment capacity of beams reinforced with BFRP bars is higher than the capacity of similar steel-reinforced concrete beams having the same reinforcement ratio. However, this is not surprising given the much higher ultimate tensile strength of BFRP reinforcing bars. However, slabs reinforced by conventional steel bars exhibited a higher moment than similar slabs reinforced with the same BFRP reinforcement ratio. This may be attributed to the development of membrane action in slabs reinforced with steel as the steel is able to yield, while BFRP will continue to carry the load until failure, without yielding.

**Figure 7:**
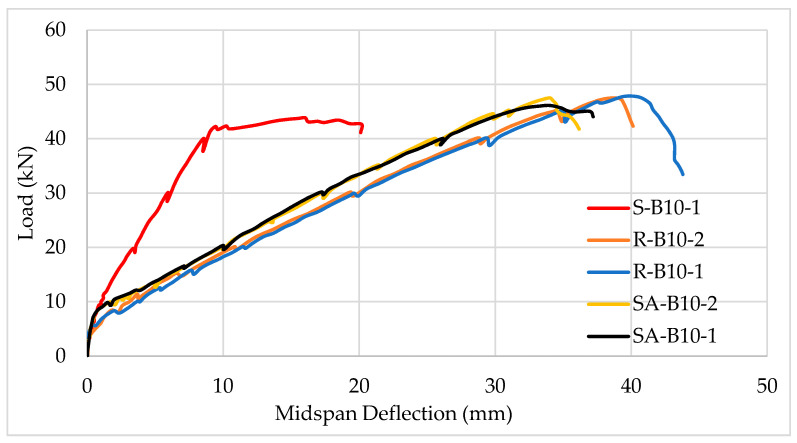
Steel-reinforced beam (S-B10-1) is much stiffer than similar BFRP-reinforced beams.

The much higher stiffness of steel reinforcing bars compared to BFRP bars increases the overall beam stiffness. The experimental study by Shamass and Cashell on the beam shown in Figure 7 confirms the reduction in beam stiffness when reinforced with BFRP bars, without decreasing the load-carrying capacity of an equivalent beam reinforced with carbon steel. Figure 7 shows that a steel-reinforced beam (S-B10-1) was much stiffer than the BFRP beams (SA-B10-2, SA-B10-1, R-B10-2, and R-B10-1).

In general, BFRP-reinforced flexural members exhibit significant cracking and large deflections, but they develop fast with a limited warning before failure [33]. The large deflections of flexural members are caused by the BFRP bars’ ability to undergo large elastic tensile deformations. However, the ductility commonly experienced in under-reinforced steel-reinforced beams is not seen in traditional BFRP-reinforced flexural members [2]. As BFRP bars will fail by sudden tensile rupture, there is no clear preference for tension-controlled concrete sections, compared to compression-controlled sections reinforced with BFRP bars. Some researchers see a marginal advantage in designing FRP sections as compression controlled by concrete crushing than FRP tensile rupture, due to the inelastic deformations associated with concrete crushing [34]. However, ultimate flexural capacity increases with an increase in flexural reinforcement, regardless of the failure mode. In all cases, whether the section is designed as tension controlled or compression controlled, the strength and serviceability requirements of the design must be met.

### 4.2. Cracking Moment and Load

The cracking moment is a property of the concrete section, which depends on the modulus of rupture and cross-sectional dimensions. It represents the moment corresponding to the initiation of flexural rupture and inception of the first crack. A fundamental assumption in its calculation is that it does not depend on the type of reinforcement, as shown in Equations (4)–(7) for the American Concrete Institute (ACI) code, Canadian code, Russian Code, and European code (EC), as well as the mechanics expression for cracking moment in Equation (8).

(4) $$f_{r,ACI}=0.62\;\sqrt{f_{c}^{\prime }}$$

(5) $$f_{r,CAN}=0.6\;\sqrt{f_{c}^{\prime }}$$

(6) $$f_{r,RUS}=0.23\;{\left(f_{cu}^{150}\right)}^{\frac{2}{3}}$$

(7) $$f_{r,EC2}=0.3\;{\left(f_{c}^{\prime }\right)}^{\frac{2}{3}}$$

(8) $$M_{cr}=\frac{f_{r}I_{g}}{y_{t}}$$

The cracking moment may also be estimated experimentally by noting the cracking load, which corresponds to the change of stiffness of the experimentally developed load-deflection curve.

An experimental study by [15] indicates that Equation (8) overestimates the cracking moment for BFRP-reinforced rectangular beams, especially when the bars are sand treated, which is consistent with the finding of [35]. The same equation slightly underestimates the cracking moment of an equivalent beam reinforced with traditional carbon steel. This phenomenon, tested by Shamass and Cashell, is consistent amongst codes, especially ACI318 [36] and the Canadian code [23]. In experimental research by [11], the experimental cracking moment of BFRP-reinforced beams was 24% to 27% lower than the values predicted using Equation (8). However, the BFRP reinforcement ratio did not correlate or affect the cracking moment. It is worth noting that BFRP beams are generally designed as over-reinforced, with a ratio reinforcement ratio larger than the balanced ratio. Similarly, experimental data by [27] shows that the experimentally measured cracking load P_cr_ for BFRP-reinforced beams was higher than that predicted values using ACI440.1R-15 or CSA, while the measured cracking load was very close to code-based predicted values for beams reinforced with traditional carbon steel.

As indicated in the previous section, studies consistently showed that cracking moments exhibited by BFRP-reinforced flexural members are higher than those predicted by various codes, although by different amounts. Similarly, the modulus of rupture increases by adding an adequate amount of basalt fibers, such as basalt macrofibers (BMFs), to the concrete mix [37]. More than 0.5% and up to 2% BMFs by concrete volume were shown to increase the modulus of rupture compared to slabs that were reinforced with BFRP without BMFs. In general, the effect of basalt fibers on fresh and mechanical properties of concrete depends on the mechanical and geometric properties of the basalt fibers themselves, in addition to concrete mix constituents and additives. Studies have shown that in the presence of fly ash, a dosage of 1% basalt fibers by volume produced optimum results for concrete strength and resistance to chloride penetration resistance, compared to higher or lower dosages [38].

### 4.3. Fatigue and Creep of BFRP Reinforcing Bars

FRP bars in general, including BFRP, have low compression capacity, therefore, their use is mostly investigated to reinforce flexural members including beams and slabs. Applications in flexural members of bridge structures are likely to require a proper understanding of fatigue response. A study by [30] on BFRP-reinforced sea sand concrete beams proposed a threshold load level of 0.55f_u_. ACI440.1R-15 [2] sets a much more stringent fatigue stress limit of 0.2f_u_ for structural elements reinforced with GFRP bars, and 0.3f_u_ for AFRP bars. However, a limit of 0.55f_u_ is set by ACI441.1R-15 for CFRP bars, which is similar to the recommendation of given by [30] for BFRP bars. Fatigue stress is proportional to the service sustained and fatigue loads and is calculated using Equation (9).

(9) $$f_{fs,sus}=M_{s,sus}\;\frac{n_{f}d\left(1-k\right)}{I_{cr}}\;$$

where

$M_{s,sus}$: the maximum service (unfactored) moment due to all sustained and fatigue loads combined;
k: the ratio of neutral axis depth to effective depth;
$I_{cr}$: the cracked moment of inertia.

(10) $$I_{cr}=\frac{bd^{3}}{3}k^{3}+n_{f}\;A_{f}\;d^{2}\;{\left(1-k\right)}^{2}$$

(11) $$k=\sqrt{2\;\rho_{f}\;n_{f}+{\left(\rho_{f}\;n_{f}\right)}^{2}\;\;}-\rho_{f}\;n_{f}$$

Additionally, the reinforcement ratio of FRP bars is defined as

(12) $$\rho_{f}=\frac{A_{f}}{bd}$$

Sustained loads over an extended period of time cause progressive deformation in FRP bars, known as creep. Flexural members subjected to bending due to various external loads, including sustained loads, will induce tensile stresses in reinforcing FRP bars. After a period of time, known as endurance time, sustained tensile stresses and resulting creep can cause a failure in FRP bars known as creep rupture. When stresses are sustained, a reinforcing BFRP bar will fail at values much lower their ultimate strength of BFRP bars. It is therefore important to control the tensile stress level in FRP bars. Creep of BFRP bars is complex and affected by the properties of fibers, resin, and the interface between fiber and resin, and it is often assumed with reasonable accuracy that the creep of FRP bars is dominated by the resin properties [39]. It is for the most part the susceptibility of the resin to creep that makes FRP bars have a lower creep–rupture threshold compared to traditional steel bars.

The most suitable mathematical model relating the ratio of the creep stress at failure/the initial ultimate strength to the sustained load duration (time) is found to be a linear relationship with logarithmic time [40,41,42].

### 4.4. Nominal Flexural Capacity 

When FRP reinforcing bars rupture, failure of the reinforced flexural member is catastrophic, regardless of the fiber used (carbon, glass, aramid, or basalt). Compared to steel-reinforced flexural members, FRP-reinforced beams offer a limited warning of impending failure in the form of extensive cracking and large deflections. These large deflections of the concrete membrane at failure are caused by the large elastic elongation of FRP bars before their rupture. This is especially true when the section is designed as tension controlled where failure is controlled rupture of FRP bars. In addition, the much smaller modulus of elasticity of the FRP reinforcing bars also contributes to deflections that are much larger than an equivalent steel-reinforced flexural member.

The addition of 43 mm long basalt microfibers (BMFs) having 1000 MPa tensile strength tends to increase the stiffness of concrete slabs reinforced with BFRP bars when the volume fraction of BMFs exceeds 0.5%. This is demonstrated by a decrease in deflection of over-reinforced ($\rho_{f}=1.4\;\rho_{fb}$ and $\rho_{f}=2.8\;\rho_{fb}$) BFRP-reinforced slabs as the volume fraction of BMFs increases from 0.5% to 2.00% [37].

**Figure 8:**
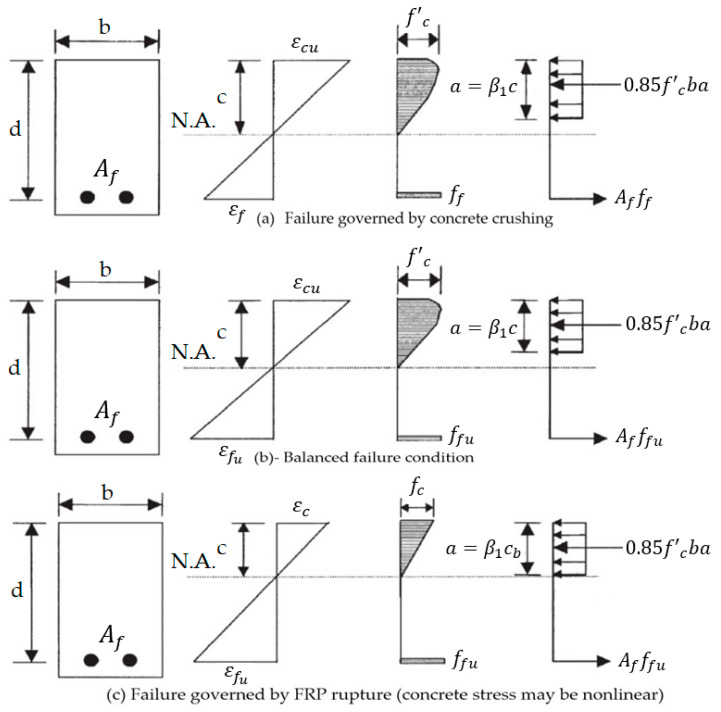
Strain and stress distribution at ultimate conditions [2].

ACI440.1R-15 uses a design philosophy for concrete flexural members reinforced with BFRP similar to the philosophy adopted by ACI318 [43], which recognizes the differences between FRP bars and traditional carbon steel bars, such as the fact that FRP bars do not yield but fail by tension rupture. At ultimate conditions, whether the flexural member fails by concrete crushing or FRP rupture, concrete reaches the ultimate compressive strength of $\varepsilon_{cu}=0.003$, as shown in Figure 8. Failure of the flexural member is controlled by tension rupture of FRP bars when the reinforcement ratio $\rho_{f}$ is less than the balanced reinforcement ratio $\rho_{fb}$. When tension rupture controls, as shown in Figure 8c, the nominal flexural capacity, $M_{n}$, for a section with an area of FRP bars, $A_{f}$, is given by Equation (13).

(13) $$M_{n}=A_{f}f_{fu}\;\left(d-\frac{\beta_{1}c}{2}\right)$$

The design tensile strength $f_{fu}$, given by Equation (14), considers the long-term effects of the environment (such as marine and alkaline environments) on degrading the tensile strength.

(14) $$f_{fu}=C_{E}\;f_{fu}^{*}$$

where:

$f_{fu}^{*}$ = guaranteed tensile strength of FRP bars, defined as the mean tensile strength of a sample of test specimen minus three times standard deviation ($f_{fu}^{*}=f_{fu,\;average}-3\sigma )$, MPa (psi).

The environmental reduction factor, $C_{E}$, is taken as 0.8 or 0.7 [2] depending on the severity of exposure conditions for FRP bars made of glass fibers, while the factor is 0.9 or 1.0 for carbon fibers. No data are provided for BFRP bars, but current research is covered in a subsequent section of this article.

## 5. Shear Strength and Response of BFRP-Reinforced Beams and Transverse Shear of Bars

Unlike conventional reinforcing steel bars, FRP bars are anisotropic, characterized by high tensile strength in the direction of fibers. This affects the shear strength and dowel action of FRP bars [2]. Transverse shear resistance of FRP bars is relatively low and largely dominated by the polymer, which negatively impacts its contribution to the shear resistance of the entire concrete reinforced with FRP bars. A test procedure for estimating the transverse shear of the FRP bars, in general, is described by [44].

Most design codes and standards consider the shear capacity of a concrete cross-section, *V_n_*, to be the combination of shear resistance provided by concrete mechanisms, *V_c_*, and shear resistance provided by the reinforcing FRP stirrups, *V_f_*. Studies have shown that the shear capacity of concrete, *V_c_*, is influenced by the axial stiffness of tension reinforcement (product of the modulus of elasticity times the tension reinforcement area) [45]. Other studies on beams reinforced by BFRP bars also indicate that with or without BFRP shear reinforcement, the load at which the first diagonal shear crack occurs increases with the flexural reinforcement ratio [16]. Despite the high ultimate tensile strength *f_u_*, the axial stiffness of FRP bars is lower than steel bars of the same area, due to the lower modulus of elasticity of FRP bars in general. As a result of the relatively lower axial stiffness, the neutral axis depth of the cracked FRP-reinforced concrete section is shallower than an equivalent concrete section with an equal area of reinforcing steel. As a result, ACI440.1R-15 recommends the concrete contribution to shear resistance given by Equation (15), which depends on the neutral axis depth *kd*. The neutral axis depth *kd* is dependent on the ratio of FRP reinforcement ratio, $\rho_{f}$, and the modular ratio, $n_{f}$.

(15) $$V_{c}=\frac{2}{5}\;\sqrt{f_{c}^{\prime }\;b_{w}\;\;}\;\left(kd\right)$$

(16) $$k=\sqrt{2\;\rho_{f}\;n_{f}+{\left(\rho_{f}\;n_{f}\right)}^{2}\;\;}-\rho_{f}\;n_{f}\;$$

where

$\rho_{f}$ = fiber-reinforced polymer reinforcement ratio;
$n_{f}$ = ratio of modulus of elasticity of FRP bars to the modulus of elasticity of concrete;
$V_{f}$ = shear resistance provided by FRP stirrups, N (lb);
$V_{n}$ = nominal shear strength at section, N (lb);
$V_{s}$ = shear resistance provided by steel stirrups, N (lb);
$V_{u\;}$ = factored shear force at section, N (lb);
w = maximum allowable crack width, mm (in);
$f_{fv}$ = tensile strength of FRP for shear design, taken as smallest of design tensile strength;
$f_{fu}$ = the strength of the bent portion of FRP stirrups $f_{fb}$, or stress corresponding to $0.004E_{f}$, MPa (psi);
$\rho_{fv}$ = ratio of FRP shear reinforcement.

Noting the effect of the relatively low axial stiffness of FRP bars in general compared to steel reinforced concrete members on shear strength, it is therefore beneficial to consider increasing the reinforcement ratio and design concrete flexural members as over-reinforced. Increasing the reinforced ratio and/or modulus of elasticity of BFRP was shown to reduce shear crack width and increase the contribution of uncracked concrete to shear resistance by increasing the depth of the compression block and aggregate interlock [46].

BFRP shear reinforcement placed perpendicular to the axis of the member is effective in resisting shear failure and increasing the load-carrying capacity, especially when the beam is tension controlled [16]. BFRP shear reinforcing stirrups also increase resistance to shear failure in compression-controlled beams, but to a lesser extent compared to tension-controlled beams. As shear rupture of the stirrups is common when beams are reinforced with BFRP stirrups, ACI440.1R-15 places a strict limit on the stress in stirrups, $f_{fv}$, as given by Equation (17). The strict stress limit also controls crack widths, which are wider in FRP-reinforced beams, and ensures the integrity of the beam by avoiding failure at the bent portion of the FRP stirrups. Such a limit, however, needs investigation for BFRP stirrups as ACI4410.1R-15 does not explicitly address reinforcing bars made of basalt fibers.

(17) $$f_{fv}=0.004\;E_{f}\leq f_{fb}$$

where

*f_fb_* = strength of the bent portion of FRP, MPa (psi).

The sheer force carried by the stirrups is proportional to the stress, $f_{fv}$, in FRP stirrups, longitudinal spacing of FRP stirrups along the beam, s, and area of the vertical legs of stirrups, $A_{fv}$. For CFRP, AFRP, and GFRP, ACI4401.R-15 adopts the mechanics-based Equation (18), which will likely remain the same for BFRP stirrups when incorporated in the standard.

(18) $$V_{f}=\frac{A_{fv}f_{fv}d}{s}$$

In seismic areas, where the use of lightweight concrete is of interest, studies have shown that BFRP-reinforced beams exhibited the same concrete contribution to shear resistance as that of normal-weight concrete [46]. That contribution to shear resistance was also the same for lightweight concrete and normal-weight concrete, whether BFRP bars were sand coated without helical wrapping, or helically wrapped without sand coating.

## 6. Durability

It is important to develop a reasonable understanding of the long-term structural response of concrete members reinforced with BFRP bars in an unfavorable environment. The unique structure of FRP bars, consisting of fibers and resin, makes it important to evaluate their durability in aggressive environments such as prolonged exposure to elevated temperature, highly alkaline environments, and freezing/thawing and low temperature. In addition, moisture ingress into the resin, before placement in concrete, could lead to degradation of mechanical properties of FRP reinforcing bars. Some studies indicate that vinyl ester resin offers better resistance to moisture ingress compared to other types of resins used in making FRP bars. Studies have shown that up to 40% of the tensile strength of GFRP bars can be lost after exposure to a combination of ultraviolet rays and moisture tests with and without loading [47]. Tensile tests of GFRP, BFRP, and CFRP bars, made of vinyl ester resin, conducted after immersion in tap water (pH = 7.00) for 180 days, showed various degrees of degradation [48]. BFRP and CFRP bars retained nearly 89% of the tensile strength, while GFRP retained 78%. Other studies confirmed the superior performance of vinyl ester resin in terms of moisture uptake, where BFRP bars made with vinyl ester resin exhibited lower moisture uptake (40%) compared to BFRP bars made with epoxy resin (68%) [49]. The moisture uptake measurements were done after conditioning the BFRP bars in an alkaline solution for 5000 h at a temperature of 60 °C.

### 6.1. Properties of BFRP Bars in Relatively Elevated Temperature 

The bond strength between ribbed BFRP bars (without wrapping or sand coating) and concrete appears to improve in samples subjected to higher temperatures of 50 °C and 60 °C for 1.5 months, compared to samples tested after 1.5 months of exposure to 40 °C [50]. The reasons for such an increase are not clear, but researchers hypothesized that an increase in concrete strength at a higher temperature may be the cause of the relatively enhanced bond strength. However, bond strength decreased by 16% for samples immersed in alkaline solution for 6 months at 40 °C. The reduction in bond strength for similar samples subjected to higher temperatures of 50 °C and °C was lower (7% and 5%, respectively), confirming the positive effect of relatively elevated temperature on bond strength. 

### 6.2. Effect of Alkaline Environment on Properties of BFRP Bars

Alkalinity is defined as the condition of having or containing hydroxyl ions (OH^−1^) [2]. In experimental studies, alkaline solutions may be prepared using calcium hydroxide, potassium hydroxide, sodium hydroxide, etc. [51]. Aqueous solutions with pH ranging from 11.5 to 13 are known to degrade the tensile strength and stiffness of GFRP reinforcing bars [52]. It is necessary to examine the research findings on the effect of such an aggressive environment on the properties of BFRP bars.

Engineering properties of BFRP bars themselves are adversely impacted by the alkaline environment, especially at temperatures higher than 40 °C. Alkaline environments with pH values between 8 and 10 are likely for mortar and concrete during service life [53]. A pH of up to 13 may occur in an aggressive alkaline environment. The deterioration in properties is mostly due to the disintegration of the matrix, which accelerates at relatively high temperatures. Some of the properties that deteriorate after exposure to an alkaline environment include transverse shear strength, interlaminar shear strength, and flexural strength. The method in [54] is one of the tests to evaluate the resistance of FRP bars to deterioration of properties caused by an alkaline environment. ASTM D7705 recognizes the effect of moderately high temperature in accelerating the matrix deterioration in an alkaline environment by specifying a test temperature of 60 °C. It was noted that shear strength and interlaminar shear strength of larger bar diameters are less impacted by alkaline environments than smaller diameters. The higher strength retention in larger diameter BFRP bars is attributed to the smaller affected thickness [20]. On the other hand, the tensile strength retention of smaller BFRP bar diameters after exposure to an alkaline environment was higher than larger bar diameters. Al Rifai et al. [55] showed that BFRP bars lost 29% of their original tensile strength after 9 months of conditioning in an alkaline environment at 60 °C. Elgabbas et al. reported that after 3 months of conditioning in alkaline solution, BFRP bars lost 21.2% of the ultimate tensile strength [10]. That study found that the same BFRP bars, with 77.4% fiber content by weight, lost approximately 5% of the modulus of elasticity. The stability of the modulus of elasticity was also observed in 6 mm diameter BFRP bars made with epoxy resin and conditioned for 63 days in alkaline solution, saline solution, or even acid solution [56]. Examination under a scanning electron microscope (SEM) showed that the degradation of mechanical properties of these BFRP bars made with vinyl ester resin occurred at the fiber–matrix interface rather than in the matrix or fiber [10]. It is therefore clear that BFRP bars’ loss in the modulus of elasticity is limited after exposure to various aggressive environments. 

Conditioning in alkaline solution (pH = 12.8) at a relatively high temperature of 60 °C for 5000 h (7 months) caused a loss of 8.3% in the modulus of elasticity of BFRP bars [51]. The fiber content of 20 mm diameter BFRP bars used in this study was 81%, impregnated with vinyl ester resin through pultrusion.

Studies by [57] showed that CFRP bars offered the highest tensile strength retention after conditioning in an alkaline environment for three months at 60 °C, followed by BFRP bars, then GFRP bars. However, the authors found that the alkali resistance of different FRP bars is sensitive to the type of resin, fiber, and manufacturing method. For instance, GFRP with vinyl ester resin was found to exceed the 80% minimum retention of tensile strength and inter-laminar shear stress after conditioning in an alkaline environment. GFRP bars made with E-glass fibers, in particular, exhibited the highest inter-laminar shear strength retention after exposure to an alkaline environment, compared to BFRP and CFRP. Alkaline resistance of BFRP bars made of polyurethane and epoxy resins is better than alkaline resistance when vinyl ester resin is used [57].

Prediction models for ultimate tensile strength and moduli of BFRP bars in an alkaline concrete/mortar environment estimate that after 100 years of exposure at temperatures up to 60 °C, 72% of the ultimate tensile strength and 80% of the modulus of elasticity are retained [53]. The BFRP bars were ribbed and sand coated (0.5 mm coating thickness), manufactured using epoxy resin, and included diameters from 3 mm to 10 mm.

**Figure 9:**
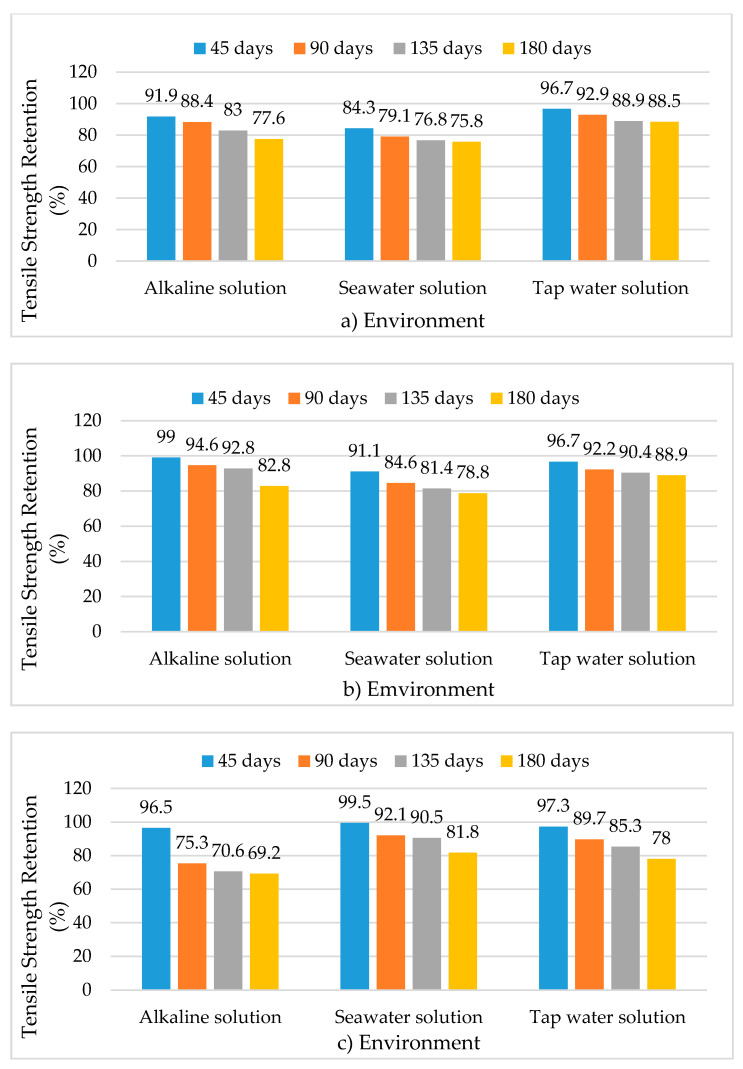
Tensile strength retention in 8 mm diameter FRP bars made of vinyl ester matrix subjected to three environments with bars made of (**a**) BFRP fibers, (**b**) CFRP fibers, (**c**) GFRP fibers. Reprinted with permission from ref. [48]. Copyright 2021 American Society of Civil Engineers

Room temperature (20 °C ± 2 °C) studies on 8 mm diameter bars made of vinyl ester matrix that were immersed in alkaline solution (pH = 12.9) for 180 days show the relative superiority of BFRP bars compared to GFRP in terms of retention of tensile strength. BFRP bars retained 77.6% of the unconditioned tensile strength while GFRP bars retained 69.2%. CFRP experienced the least deterioration in the same alkaline environment by retaining 82.8% of the unconditioned tensile strength [48], the same percentage of tensile strength after immersion in seawater solution for 180 days at room temperature. Figure 9 shows the tensile strength retention percentages of 8-mm bars after immersion in alkaline solution (pH = 12.9) seawater and tap water. GFRP bars in vinyl ester matrix are particularly vulnerable to moisture compared to BFRP and CFRP as immersion in tap water for 180 days leads to the lowest retention of tensile strength.

Experimental studies conducted to date have produced data on bond durability with significant scatter in terms of the loss of bond strength due to conditioning. Such scatter is caused by research campaigns being designed with different goals set by researchers. As a result, bond durability models that take advantage of the abundant test data have proved to be challenging [58].

### 6.3. Response of BFRP-Reinforced Concrete Members Subjected to Freezing and Thawing Cycles and Low Temperature

The performance of BFRP-reinforced concrete structures at low temperatures and subjected to freezing and thawing cycles is also of interest to researchers and designers. Exposure to low temperature and freezing/thawing (FT) cycles did not change the bond failure mode in experimental studies [59]. Up to a freezing temperature of −20 °C, the failure mode in the bond test was dominated by shear between BFRP bars and concrete. However, the bond strength at −20 °C decreased by 10% compared to original samples tested under normal temperature, without exposure to FT cycles. Studies on the effects of FT cycles on bond strength were conflicting. Some studies indicate that FT cycles of 100 and 200 (two cycles per day) had a limited effect on the bond strength [59], while other studies indicate a reduction in bond strength after samples were subjected to FT cycles (10 cycles) [60]. The effect of FT cycles on bond strength needs further investigation.

## 7. Conclusions

This article reviewed the current state-of-knowledge on research related to the application of basalt fiber-reinforced polymer (BFRP) as reinforcing bars for concrete. The review emphasized mechanical properties and durability as being the most critical for engineers in professional practice as well as researchers. The most commonly researched and used fibers for FRP bars are glass, carbon, and aramid, therefore, FRP bars with these materials have been incorporated in international design guides and standards on concrete reinforced with FRP bars, such as ACI440.R-15. The use of basalt fibers to produce concrete reinforcing FRP bars is gaining popularity due to their competitive durability, natural corrosion resistance, nonmagnetic properties, and sufficiently high tensile strength. Although carbon steel retains its environmental advantage of being 100% recyclable, its manufacturing process continues to emit significant CO_2_ into the atmosphere. The major findings of this review include:

- Similar to all FRP reinforcing bars, the BFRP stress–strain relationship is linear until failure by tensile rupture, unlike traditional reinforcing steel which reaches yield stress, becomes inelastic, strain hardens to ultimate strength, and then softens to rupture.
- Characteristics of the bond between BFRP bars and the surrounding concrete are influenced by factors including rib height and rib spacing. Many studies confirm that the most effective bond strength is achieved when BFRP reinforcing bars are ribbed, helically wrapped, and sand coated. One study reported that sand-coated ribbed bars achieved better bond strength than helically wrapped and sand-coated bars. The bond coefficient of helically wrapped and sand-coated BFRP bars was found to be comparable, and sometimes superior, to the bond coefficient of traditional carbon reinforcing steel. The bond coefficient is used in design codes to control crack width, an essential serviceability consideration of FRP-reinforced flexural concrete members. Loss of bond strength of BFRP bars at elevated temperatures is equivalent to that of GFRP bars. However, at an elevated temperature of 350 °C, a significant loss of bonding occurs in BFRP bars.
- The modulus of elasticity of FRP bars is much lower than traditional steel bars, leading to higher deflections in equivalent FRP-reinforced flexural members. As a result, the over-reinforced design offers the relative advantage of reduced deflections compared to under-reinforced FRP flexural members. Nonetheless, most standards, including ACI4401.R-15, provide guidance on designing FRP-reinforced beams as under-reinforced or over-reinforced.
- Experimental studies confirmed that tensile strength, shear strength, and interlaminar shear strength deteriorate over time when BFRP bars are conditioned in alkaline solution, especially at high temperatures. At a relatively high temperature of 60 °C, BFRP bars were found to lose nearly 29% of the tensile strength after 9 months of conditioning in alkaline solution. Due to the larger exposure area, larger bar diameters were more impacted by the alkaline environment than smaller diameters. Deterioration in the modulus of elasticity was much smaller than in tensile strength.
- There is a need for standardization of the manufacturing of BFRP bars with the goal of providing reliable guaranteed mechanical properties, such as tensile strength and shear strength. Significant variability is reported in mechanical properties, often including bars produced by the same manufacturer. One reason for the variability in properties is the inconsistency in the application method and composition of fiber size, a thin layer of treatment applied to the fibers during manufacturing. Currently, pultrusion is the most common method for manufacturing BFRP bars.
- Future research needs: Limited research is available on the structural response of cast-in-place (CIP) BFRP-reinforced concrete members under compression and/or combined compression and flexure, which currently restricts the application of BFRP reinforcement to flexural members. Further research is needed to quantify the effect of rib depth and rib spacing of deformed BFRP bars on the structural response of BFRP-reinforced flexural members. Sand coating to enhance bonding of BFRP to the surrounding concrete is still an open research area in terms of effectiveness and clarity of specifications of the materials and methods of application.

## Abbreviations

**Variable**	**Description**
BFRP	Basalt Fiber-Reinforced Polymer
*k_b_*	Bond Coefficient
FRP	Fiber-Reinforced Polymer
GWP	Global Warming Potential
MRI	Magnetic Resonance Imaging
GFRP	Glass Fiber-Reinforced Polymer
CFRP	Carbon Fiber-Reinforced Polymer
AFRP	Aramid Fiber-Reinforced Polymer
HCC	Hollow Concrete Column
*f_u_*	Ultimate Stress
*f_v_*	Allowable Stress
$\;\varepsilon_{cu}$	Ultimate Strain
*r_s_*	Rib Spacing
*r_h_*	Relative Rib Area
*w_c_*	Concrete Lug Width
*w_f_*	FRP Bar Lug Width
τ	Average Bond Stress
F	Tensile Force
$C_{b}$	Effective Circumference of FRP Bar
l	Bonded Length
$S_{max}$	Maximum Spacing Between Tension Reinforcing FRP Bars.
$E_{f}$	Design or Guaranteed Modulus of Elasticity of FRP Defined as Mean Modulus of Sample of Test Specimen $(E_{f}=E_{average}$)
$f_{fs}$	Stress Level Induced in FRP at Service Load
$C_{c}$	Clear Cover
w	Maximum Crack Width on the Tension Side
$E_{f\;}$	Modulus of Elasticity of FRP
$F_{f\;}$	Flexural Stress in FRP Bars
β	Ratio of the Distance from the Neutral Axis to Extreme Tension Fiber to Distance from Neutral Axis to Center of Tensile Reinforcement
$d_{c}$	Thickness of Concrete Cover Measured from Extreme Tension Fiber to Center of Tension Bars
s	Spacing of Longudinal Bars
$M_{n}$	Nominal Flexural Capacity
$f_{r,ACI}$	Fracture Stress (American Concrete Institute)
$f_{r,CAN}$	Fracture Stress (Cadian Code)
$f_{r,RUS}$	Fracture Stress (Rsian Code)
$f_{r,EC2}$	Fracture Stress (Eopean code)
$M_{cr}$	Cracking Moment
$f_{fs,sus}$	Fatigue Stress due to Sustned and Fatigue Load
$n_{f}$	Ratio of Modulus of Elasticity of FRP Bars to the Modulus of Elasticity of Concre
d	Effective Depth
k	Ratio of Neutral Axis Deh to Effective Depth
$I_{cr}$	Cracked Moment of Inertia
$A_{f}$	Area of FRP Bars
$\rho_{f}$	Fiber-Reinforced PolymeReinforcement Ratio
b	Width
$f_{fu}$	Design Tensi Strength
$f_{fu}^{*}$	Guaranteed Tenle Strength
$C_{E}$	Environmental Reduction Factor
V_n_	Nominal Shear Strength at Section
V_c_	Shear Resistance Provided by Concrete Mechanisms
V_f_	Shear Resistance Provided by the Reinforcing FRP Stirrups
$V_{s}$	Shear Resistance Provid by Steel Stirrups
$V_{u\;}$	Factored Shear Foe at Section
w	Maximum Allowab Crack Width
$f_{fv}$	Tensile Strength of FRP for Shear Design
$\rho_{fv}$	Ratio of FRP Shear Reinforcement
$f_{fb}$	Strength of the Bent Portion of FRP
$A_{fv}$	Area of FRP Stirrups
s	Spacing between Stirrups
